# IL-17A promotes tumorigenesis and upregulates PD-L1 expression in non-small cell lung cancer

**DOI:** 10.1186/s12967-023-04365-3

**Published:** 2023-11-17

**Authors:** Hua Liao, Xiaodan Chang, Lin Gao, Cuiping Ye, Yujie Qiao, Lingyan Xie, Jie Lin, Shaoxi Cai, Hangming Dong

**Affiliations:** 1 Chronic Airways Diseases Laboratory, Department of Respiratory and Critical Care Medicine, Nanfang Hospital, Southern Medical University, Guangzhou, China; 2 Department of Respiratory and Critical Care Medicine, The Fifth Affiliated Hospital, Southern Medical University, Guangzhou, China; 3 School of Nursing, Southern Medical University, Guangzhou, Guangdong China; 4 Department of Pathology, School of Basic Medical Sciences, Southern Medical University, Guangzhou, Guangdong China

**Keywords:** Interleukin-17A, NSCLC, Autophagy, PD-L1, Immunotherapy

## Abstract

**Background:**

The tumor microenvironment plays a key role in non-small cell lung cancer (NSCLC) development and also influences the effective response to immunotherapy. The pro-inflammatory factor interleukin-17A mediates important immune responses in the tumor microenvironment. In this study, the potential role and mechanisms of IL-17A in NSCLC were investigated.

**Methods:**

We detected IL-17A by immunohistochemistry (IHC) in 39 NSCLC patients. Its expression was correlated with the programmed cell death-ligand1 (PD-L1). IL-17A knockdown and overexpression in A549 and SPC-A-1 cell models were constructed. The function of IL-17A was examined in vitro by wound healing, migration, invasion, plate colony formation and T cell killing assay. Western blot analysis, immunofluorescence assay and IHC were performed to investigate the regulation effects of IL-17A on autophagy in A549 and SPC-A-1. The effect of IL-17A on ROS/Nrf2/p62 signaling pathway was detected. Subcutaneous tumor models were established to examine the tumor-promoting effect of IL-17A in vivo and its effect on immunotherapy.

**Results:**

We found a prevalent expression of IL-17A in NSCLC tumor tissues and it was positively correlated with PD-L1 expression (r = 0.6121, p < 0.0001). In vitro, IL-17A promotes lung cancer cell migration, invasion and colony formation ability. Moreover, IL-17A upregulated N-cadherin, Twist, and Snail, and downregulated E-cadherin in NSCLC cells. IL-17A enhanced cell survival in the T cell killing assay. Mechanistically, IL-17A induced ROS production and increased Nrf2 and p62 expression, thereby inhibiting autophagy and reducing PD-L1 degradation. In vivo experiments, anti-IL-17A monoclonal antibody alone slowed the growth of subcutaneous tumors in mice. When combined with anti-PD-L1 monoclonal antibody, tumor tissue expression of PD-L1 was reduced and the therapeutic effect was diminished.

**Conclusion:**

We found that IL-17A promoted NSCLC progression and inhibited autophagy through the ROS/Nrf2/p62 pathway leading to increased PD-L1 expression in cancer cells. Modulation of IL-17A may affect the therapeutic efficacy of immunotherapy.

## Introduction

Lung cancer is one of the most prevalent cancers worldwide, accounting for 11.4% of the total incidence of all cancers and the leading cause of cancer deaths, accounting for 18.0% of all cancer deaths [1]. Among the common subtypes of lung cancer, NSCLC accounts for 85% of the total cases [2], and its treatment is of great interest. Recent advances in molecular testing technologies have led to a better understanding of the genomic mutations that cause NSCLC and the subsequent development of new drugs. Despite the tremendous advances in cancer treatment over the past decades, resistance to classical chemotherapeutic agents, novel targeted therapies and immunotherapeutic agents still limits the success of cancer treatment [3, 4]. The high incidence and mortality rates of lung cancer impose a serious family and economic burden on patients and their families, and there is an urgent need to find more effective treatments.

Immunotherapy is a hot topic among many tumor treatment approaches. Immune checkpoint inhibitors (ICIs) act on the inhibitory pathways of tumor immune response, restoring immune cell function to recognize and maintain anti-tumor immunity. ICIs are available to target different pathways, including programmed cell death 1 (PD-1) pathway, programmed cell death ligand 1 (PD-L1) pathway, and cytotoxic T lymphocyte antigen 4 (CTLA) pathway [5].

Multiple clinical trials have confirmed that the available immune checkpoint inhibitors, such as PD-1 inhibitors nivolumab, pembrolizumab, and PD-L1 inhibitor atezolizumab, are significantly superior to standard chemotherapy in terms of efficacy and safety [6–8]. Although PD-1 or PD-L1 immune checkpoint inhibitor therapy significantly improves the survival rate of lung cancer patients, only about 20–40% of NSCLC patients benefit from them, with a minority of patients showing durable responses to treatment, suggesting innate or acquired resistance to immunotherapy [9].

Mechanistically understanding the regulatory pathways of PD-L1 protein expression and stability can provide a molecular basis for improving the clinical efficiency and efficacy of PD-1/PD-L1 blockers in tumor patients. The presence of cytokines and inflammatory cells in the tumor microenvironment plays a crucial role in the outcome of the host antitumor response. Cytokines are released in response to cellular stress, injury or infection, promote the restoration of tissue homeostasis, and limit tumor development and progression. However, the secretion of cytokines in the inflammatory environment may promote tumor cell growth, inhibit apoptosis, and drive tumor cell invasion and metastasis [10].

IL-17A is a classical member of the IL-17 family of pro-inflammatory cytokines. IL-17A in the tumor microenvironment is mainly produced by CD4^+^ T cells, CD8^+^ T cells, γδ T cells and various innate immune cell populations. However, if the IL-17 response is dysregulated, it can promote immunopathology in the presence of infection or autoimmunity [11]. Transforming growth factor β(TGF-β), IL-23, and IL-1β can promote the differentiation of Th17 cells to produce IL-17A/F, which regulates cancer cell proliferation, migration, epithelial mesenchymal transition (EMT)through NF-κB pathway to promote tumor development and metastasis [12]. IL-17A promotes the expression of IL-6, G-CSF, MFG-E8, and CXCL1 in NSCLC tumor tissues to increase tumor-associated neutrophils (TANs) infiltration and reduce the number of tumor-infiltrating lymphocytes (TILs), leading to drug resistance to PD-1 inhibitors [13]. IL-17A is an important indicator of inflammation and autoimmune diseases. Clinical trials of immune checkpoint inhibitors often exclude patients with pre-existing autoimmune diseases to limit the risk of immune-related adverse events (irAE). But according to data from a clinical study, 13.5% to 24.6% of patients with lung cancer also carry a diagnosis of autoimmune disease (AID) [14]. Further clarifying the role of IL-17A in NSCLC may help to ascertain the risks and benefits of ICIs in this population.

Autophagy is a process in which misfolded proteins, damaged or aged organelles and mutated proteins in vesicles are isolated into autophagosomes and then bound to lysosomes, leading to the degradation of the isolated components and providing a major pathway for the turnover of stable and defective cellular proteins [15]. Autophagy is often observed during tumorigenesis and chemotherapy, and some studies suggest that autophagy plays a protective role against tumor cells during chemotherapy, leading to drug resistance and refractoriness of cancer [16].

In recent years, complex and controversial evidence for the role of autophagy in tumorigenesis has emerged. Autophagy may play a protective role against cancer by eliminating damaged organelles and recycling degradation products in normal cells. Paradoxically, excessive autophagy can lead to "autophagic cell death" or "type II programmed cell death" in cancer cells. Autophagy induced by metabolism and stress can play a pro-death or pro-survival role. Autophagy also plays a dual role in tumorigenesis, tumor progression and resistance of cancer cells to chemotherapy [17]. In the long-term chemotherapy of tumor, multidrug resistance (MDR) may also occur. Autophagy is a double-edged sword for MDR tumors: it is involved in MDR development and protects cancer cells from chemotherapy, but it can also kill MDR cancer cells which with inactive apoptotic pathways. Autophagy induced by anticancer drugs can also activate apoptosis signaling pathway in MDR cells and promote MDR reversal [18]. With the widespread application of immunotherapy, the phenomenon of immunotherapy resistance is gradually increasing. In a study of drug resistance in gastric cancer, autophagy was found to regulate PD-L1 expression in gastric cancer through the p62/NF-κB pathway. After the use of autophagy inhibitors, the expression of PD-L1 in gastric cancer cells was significantly increased, while the expression of PD-L1 was down-regulated after autophagy induction. Autophagy regulates the expression of PD-L1 in tumor cells, which may affect the efficacy of PD-L1 blockers [19].

The relationship between IL-17A and autophagy in lung cancer cells has been rarely studied. Studies related to airway remodeling in bronchial asthma suggest that IL-17A can promote fibrosis development by regulating autophagy level of bronchial fibroblasts [20]. Studies on diabetes and age-related aging suggest that increased IL-17A can inhibit autophagy, and enhanced autophagy with metformin can restore mitochondrial function and reduce aging-related inflammation [21]. All relevant studies suggest a close relationship between IL-17A and cellular autophagy.

In the clinical treatment of NSCLC, the choice of immunotherapy needs to be based on the patient's genetic testing, and pathological findings. PD-L1 is currently the most widely validated, used and accepted biomarker, expressed by pathological tissue tumor proportional score (TPS) or tumor cell (TC), defined as the percentage of tumor cells with partial or complete membranous PD-L1 staining relative to all tumor cells in the sample is used to guide patients’ selection of anti-PD-1 or anti-PD-L1 inhibitors, with patients with high TPS or TC scores more likely to benefit from ICIs treatment [22]. In immunotherapy studies for rectal colon cancer, IL-17A was shown to increase PD-L1 expression via the p65/Nrf2/miR-15b-5p axis, helping tumor cells to undergo immune escape, and blocking IL-17A improved the efficacy of anti-PD-L1 [23].

Inflammation, infection, stress and other factors can induce the increase of IL-17A. Aiming at this proinflammatory factor, we performed IHC on lung cancer tissues and paracancerous tissues of NSCLC patients. The results suggested that cancer cells were highly expressing IL-17A. Combined with relevant studies and our experimental results, we proposed the scientific hypothesis that IL-17A may be involved in the development of lung cancer in NSCLC by modulating tumor cell autophagy, altering the tumor microenvironment, improving PD-L1 expression in cancer cells, and promoting local immune cell exhaustion and immune escape of cancer cells. Targeting IL-17A may improve tumor immune microenvironment and change ICIs response.

## Materials and methods

### Cell culture

The human NSCLC cell lines A549 and SPC-A-1, and the mouse NSCLC cell line Lewis lung cancer cell (LLC) were maintained in our laboratory. Jurkat T cells were purchased from Procell Life Science & Technology Co (Wuhan China). A549 and Jurkat T cells were cultured in RPMI-1640 (Gibco, USA) with 10% fetal bovine serum (NEWZERU, NewZealand) and 1% Penicillin–Streptomycin (Gibco, USA). SPC-A-1 and LLC were cultured in DMEM (Gibco, USA) with 10% FBS and 1% Penicillin–Streptomycin solution. All cells were cultured in an incubator at 37 ℃ with 5% CO_2_.

IL-17A is a secreted protein. To understand the effect of IL-17A on NSCLC, A549 and SPC-A-1 were transfected with lentivirus to construct control, IL-17A overexpression and knockdown cell lines. A549 and SPC-A-1 cells were seeded into 6-well plates, and isotype control, overexpress and knockdown lentiviral transfection reagent were added into the 6-well plates containing 2 ml of complete normal medium when the density reached 30% fusion, using multiplicity of infection (MOI) as 10. The culture medium was refreshed after 24 h. After 72 h, 1 ng/ml puromycin was added to screen the successfully transfected cells, and the subsequent cells were continuously cultured in RPMI-1640/DMEM + 10% FBS containing 1 ng/ml puromycin.

### Mouse models of subcutaneous tumor

Approved by The Institutional Animal Care And Use Committee (ICUC-LAC-20221209-002) of Nanfang Hospital, mouse studies were carried out at the Experimental Animal Center of Nanfang Hospital. A 150 µl suspension of 1 × 10^6^ LLC cells were injected subcutaneously into the right flank of C57BL/6 mice (SPF grade, 4–5 weeks old, male). The body weight and tumor volume were measured every 3 days. The tumor volume was calculated by a formula: (length × width^2^)/2. When the tumor volume reached 50–100 mm^3^, the tumor-bearing mice were randomly divided into 4 groups (n = 5 per group): control group, anti-IL-17A group, anti-PD-L1 group, anti-IL-17A and anti-PD-L1 combined treatment group. Each mouse was treated with 100 µl PBS, 100 µg/mouse anti-IL-17A Abs (BE0173, Bioxcell, USA), 200 µg/mouse anti-PD-L1 Abs (BE0101, Bioxcell, USA), 100 µg/mouse anti-IL-17A Abs and 200 µg/mouse anti-PD-L1 Abs intraperitoneally every 3 days. Mice were sacrificed at day 18, the volume and weight of tumor tissues were recorded. Then tumor tissues were embedded for histochemical and immunohistochemistry evaluation.

### Patient samples

39 pairs of pathological sections of NSCLC tumor tissues and paracancerous tissues were collected from the Department of Pathology of Nanfang Hospital from 2020 October to 2021 August. Patients with infections and autoimmune diseases were excluded. The expression of IL-17A, LC3, and p62 protein levels were detected by immunohistochemistry analysis. All procedures in this study were carried out in accordance with the ethical protocols of the Ethics Committee of Nanfang Hospital (NFEC-2022-420, Guangzhou, China).

### Immunofluorescence assay

Transfected cells were seeded into round coverslips in 24-well plates. After 24 h, the medium was removed and cells were washed by PBS. Then cells were fixed by 4% paraformaldehyde for 30 min and then washed 3 times with PBS. Cells were blocked with 5% bovine serum albumin (BSA) for 1 h. Cells on the round coverslip were incubated with PD-L1 (66248-1, Proteintech, China), p62 (A5180, Bimake USA), LC3 (14600-1, Proteintech, China), Nrf2 (16396-1, Proteintech, China) primary antibodies overnight at 4 ℃. After being washed by PBS for 3 times, secondary antibodies (ab150077, abcam, UK, SA00013-4, proteintech, China) were added to incubate for 2 h at room temperature. Finally, cells were stained with DAPI for nuclei, coverslips were inverted on slides, and fluorescence intensity was observed by fluorescence microscopy.

### Immunohistochemical staining (IHC) assay and evaluation

Paraffin-embedded NSCLC tumor tissues and paracancerous tissues of patients and mouse subcutaneous tumor tissues were cut into 4 μm sections on slides. Heat slides for 2 h at 65 ℃ in a dry oven. Remove paraffin and rehydrate tissue by xylene and gradient alcohol. Completely submerge slides in citric-acid buffer and maintain a continuous boil for 20 min. After boiling, the slides are cooled at room temperature and washed with PBS 3 times. Cover the tissues with endogenous peroxidase blockers (PV-9001, ZSGB-BIO, China) for 10 min. Then tissues were incubated overnight with IL-17A (ab79056, abcam, UK), p62 (A5180, Bimake, USA), LC3 (14600-1, Proteintech, China), Nrf2 (16396-1, Proteintech, China), CD4 (ab288724, abcam, UK), CD8 (ab245118, abcam, UK) at 4 ℃, reaction strengthening fluid for 20 min, and secondary antibody for 2 h in room temperature. Overlay the freshly prepared DAB solution on the tissues to detect positive signals and counterstained nuclei with hematoxylin. All antibodies were diluted at the rate of 1:100. Images were captured by Olympus BX63 automatic microscope. To quantitatively analyse the difference in IL-17A expression, integrated optical density (IOD) value and area of the dyed region were detected through Image pro plus version 6.0 software. Mean density reflects the concentration per unit area of the target protein, mean density = IOD/area.

### T cell killing assay

Jurkat T cells were activated by 25 µL/ml of immunoCult™ human CD3/CD28 T cell activator (10971, STEMCELL TECHNOLOGIES, Canada)for 3 days. And then expand T cells with fresh complete ImmunoCult™-XF T Cell Expansion Medium (10981, STEMCELL TECHNOLOGIES, Canada). The transfected A549 and SPC-A-1 cells were seeded into 6-well plates at a density of 1 × 10^5^ cells per well and co-cultured with the activated Jurkat T cells at a density of 5 × 10^5^ cells per well for 48 h, then removed the medium and washed the surviving cancer cells with cold PBS. Living cancer cells were fixed by 4% paraformaldehyde for 30 min and stained with 0.1% crystal violet for 5 min.

### Western blot analysis

Disrupt the cells by RIPA lysate which contains 1% protease inhibitor, let stand for 5 min, then transfer to EP tubes, and finally centrifuge at 4 ℃ at 12,000×*g* for 15 min. Take the supernatant, add the appropriate amount of loading buffer and heat at 100 ℃ for 10 min. Separate the protein samples by SDS–polyacrylamide gel electrophoresis, then transfer them onto PVDF membranes (Merck millipore, USA), blocked with 5% BSA, and incubated with the primary antibody overnight at 4 ℃. The membranes were incubated with secondary antibodies (042-06-18-06, LICOR, USA) for 1 h and fluorescence was detected by Odyssey infrared imaging system (LICOR, USA).

### Wound healing assay

Adjust the transfected A549 and SPC-A-1 cell density and inoculate into 6-well plates overnight to reach 100% fusion. A sterile pipette tip was used to make scratches in 6-well plates. Cells were cultured using serum-free medium. The scratches area was captured at 0 h, 48 h.

### Migration and invasion assay

Transwell membranes were hydrated by basal medium. The Matrigel (356234, Corning, USA) was diluted with basal medium at a ratio of 1:9, then spread on the upper chamber surface of the transwell, and polymerized into gel state after 20 min at 37 ℃. No matrix gel was required for testing cell migration ability. Cells were starved for 24 h, digested and resuspended using serum-free medium, and taken 100 µl inoculated into the transwell at a density of 5 × 10^5^/ml. 600 µl medium containing 10% FBS was added to the lower chamber. Transwell chambers were removed, fixed in 4% paraformaldehyde methanol for 30 min, and stained with 0.1% crystal violet for 20 min. The upper chambers of non-migrated cells were gently wiped off with a cotton swab. The images were taken by a microscope and analysed by Image J.

### Plate colony formation assay

Lentivirus-transfected A549 and SPC-A-1 cells were digested, centrifuged, resuspended and counted. 1000 cells were added to each well and inoculated into a 6-well plate, which was incubated for 2 weeks at 37 ℃ in a 5% CO_2_ incubator. When the colonies were formed, the culture medium was discarded, fixed in methanol for 30 min, and stained with 0.1% crystal violet for 5 min, and eventually the colonies were photographed and counted.

### Detection of intracellular superoxide

To understand the altered expression of intracellular reactive oxygen species, intracellular levels of H_2_O_2_ and superoxide anion were measured. The transfected cells were incubated with 10 μM dihydroethidium (DHE, S0063, Beyotime, Shanghai, China) in basal medium at room temperature for 30 min. The fluorescence intensity which represents superoxide anion was detected by fluorescence microscopy. In order to detect the expression of H_2_O_2_, cells were collected into centrifuge tubes, then discard supernatant. Add lysate at the rate of 100–200 µl hydrogen peroxide assay lysate per 1 million cells, centrifuge at 12,000*g* for 3–5 min at 4 ℃, and collect the supernatant finally. Add 50 µl of sample and 100 µl of hydrogen peroxide detection reagent (S0063, Beyotime, Shanghai, China) to the assay wells. Leave at room temperature (15–30 ℃) for 30 min. Then immediately determine A560. Calculate the concentration of hydrogen peroxide in the sample from the standard curve.

### qPCR assay

Lentivirus-transfected A549 and SPC-A-1 cells total RNA was extracted using 1 ml TRIzol reagent (R411-01/02, vazyme, Nanjing, China). Chloroform was added and leave for 10 min, then centrifuge at 12,000×*g* for 15 min at 4 ℃. Pipette 400 µl of supernatant and transfer to another centrifuge tube, add equal amount of isopropanol. After 10 min of resting, centrifuge at 12,000×*g* for 10 min at 4 ℃. Pour off the supernatant, wash with 75% ethanol and blow dry. Add 20 µl DEPC H_2_O to dissolve the RNA precipitation. Reverse transcription and amplification were quantified using HiScript III All-in-one RT SuperMix (R333, vazyme, Nanjing, China) and Taq Pro Universal SYBR qPCR Master Mix (Q712-02, vazyme, Nanjing, China) kits.

### Statistical analysis

Experimental results were statistically analyzed using GraphPad Prism 9 (Inc, San Diego, CA, USA). The data were expressed as mean ± standard deviation (SD). Comparisons between groups were made using the unpaired two-tailed student's test. Data between multiple groups were compared using one-way analysis of variance (ANOVA). Patient characteristics data were tested using Fisher's exact test. Correlation between two variables was performed using Pearson Correlation Analysis. p values < 0.05 were considered as significant differences.

## Result

### IL-17A was highly expressed in NSCLC and positively correlated with PD-L1 expression

**Fig. 1:**
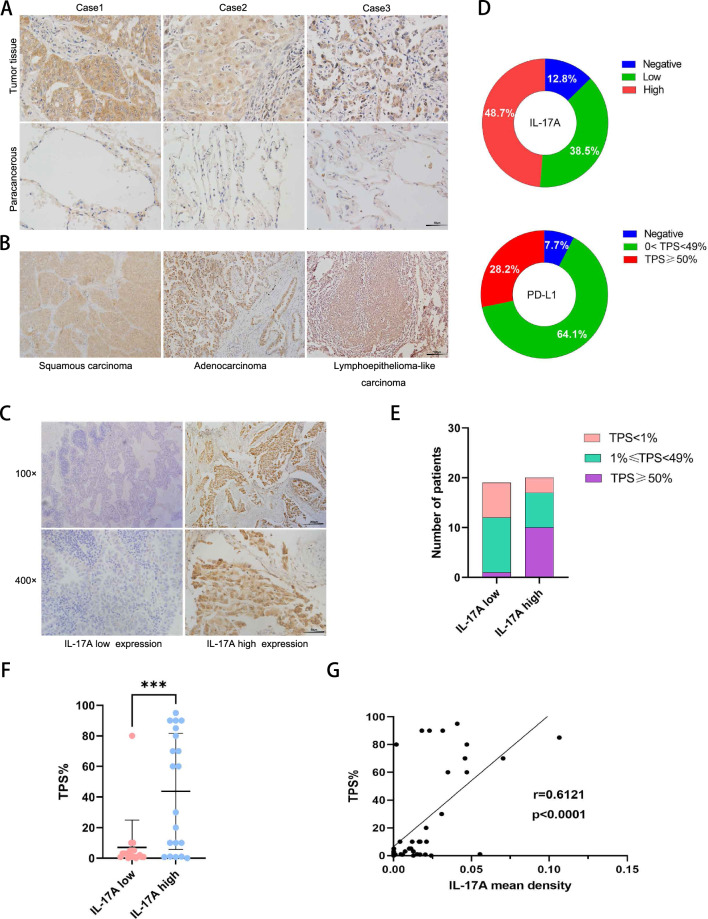
The expression of IL-17A in NSCLC positively correlates with PD-L1 expression. **A** IHC staining results of IL-17A in NSCLC tumor tissues and paracancerous tissue. The shape of the ruler in the picture is different from the other figures. **B** IHC staining results of IL-17A in NSCLC with different pathological classifications. Scale bar = 50 μm. **C** The expression of IL-17A was quantified as the mean density value (MD = IOD/area) and representative plots of the low and high expression groups. **D** Pie chart showed the proportion of low and high expression of IL-17A and PD-L1 in NSCLC patients. **E** Patients were divided into low expression group (n = 19) and high expression group (n = 20) according to the expression of IL-17A. The distribution of TPS ratio in two groups. **F** Differences in TPS scores between the two groups, p < 0.0005. **G** The correlation between IL-17A and PD-L1 expression in NSCLC tissue. r = 0.6121, p < 0.0001

To understand the role of IL-17A in NSCLC, we included tissue samples from 39 NSCLC patients. Patients with autoimmune diseases and severe infections were excluded. The tumor tissues were examined for IL-17A expression by IHC. The results showed that IL-17A expression was significantly higher in tumor tissues than in paracancerous tissues (Fig. 1A). In our collected cases, IL-17A was observed to be expressed in lung squamous carcinoma, adenocarcinoma, and lymphoepithelioma-like carcinoma, and mainly expressed in the cytoplasm and nucleus of tumor cells (Fig. 1B). We quantified the expression of IL-17A in tumor tissues using mean density values, and divided NSCLC patients into IL-17A low and high expression groups by the median of their expression level (Fig. 1C), and negative cases were classified as low expression group. The characteristic of patients’ age, gender, pathological type, smoking history, tumor size, tumor stage, and tumor proportional score (TPS) (PD-L1 IHC assays as: Dako 22C3) were also compared and analyzed. The expression of IL-17A was found to be correlated with PD-L1 (p = 0.0073, Table 1). We further analyzed the expression of IL-17A and PD-L1 in NSCLC tumor tissues, and found that the IL-17A positive rate reached 87.2% (Fig. 1D). The results revealed an increase in the proportion of patients with TPS > 1% in the IL-17A high expression group, with a particularly significant increase in the number of TPS ≥ 50% (Fig. 1E and F). We analyzed the expression correlation between these two proteins and found a positive correlation between IL-17A and PD-L1 (r = 0.6121, p < 0.0001, Fig. 1G). Therefore, we confirmed that IL-17A and PD-L1 expression in NSCLC are co-expressed.

**Table 1:** Baseline clinical characteristics of patients with NSCLC

	All patients n = 39	IL- 17A low expression n = 19	IL- 17A high expression n = 20	P value
Age (years) median (range)	61.2 (36–87)	60.8 (36–78)	61.5 (46–87)	0.840
Gender				
Male	27 (69.3)	13 (68.4)	14 (70)	0.915
Female	12 (30.7)	6 (36.6)	6 (30)	
Histology				
Adenocarcinoma	28 (71.8)	14 (73.6)	14 (70)	0.999
Squamous	11 (28.2)	5 (26.4)	6 (30)	
Smoking status				
Current/former	21 (53.9)	8 (42.1)	13 (65)	0.205
Never	18 (46. 1)	11 (57.9)	7 (35)	
Tumor size (cm)				
T1 (≤ 3)	9 (23.1)	5 (26.3)	4 (20)	0.885
T2 (> 3, ≤ 5)	18 (46.2)	8 (42.1)	10 (50)	
T3 (> 5, ≤ 7)	5 (12.8)	2 (10.5)	3 (15)	
T4 (> 7)	7 (17.9)	4 (21.1)	3 (15)	
Stage				
I	13 (33.3)	7 (36.8)	6 (30)	0.959
II	8 (20.5)	4 (21.1)	4 (20)	
III	16 (41. 1)	7 (36.8)	9 (45)	
IV	2 (5.1)	1 (5.3)	1 (5)	
TPS (%)				
≥ 50	11 (28.2)	1 (5.2)	10 (50)	0.0073**
1–49	18 (46.2)	11 (58.0)	7 (35)	
< 1	10 (25.6)	7 (36.8)	3 (15)	

The characteristic of NSCLC patients and tumor proportional score (TPS) (PD-L1 IHC assays as: Dako 22C3) were compared and analyzed. TPS: tumor proportional score. Experimental data were expressed as mean ± SD and analyzed using Fisher's precision probability test. *p < 0.05, **p < 0.01, ***p < 0.001, indicating statistically significant difference

### IL-17A promoted NSCLC cell proliferation, migration, invasion and epithelial mesenchymal transition

**Fig. 2:**
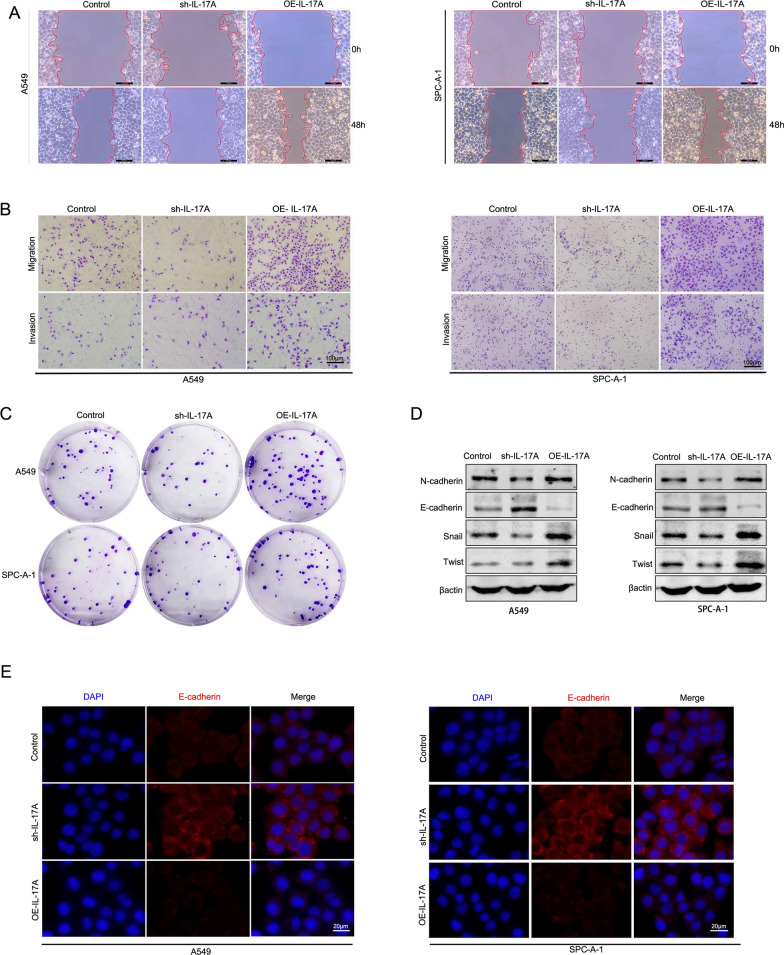
IL-17A could promote NSCLC cell proliferation, migration, invasion and EMT. **A** Wound healing assay was performed to evaluate the migration capacity of A549 and SPC-A-1 after IL-17A knockdown or overexpression. Scale bar = 100 µm. **B** Detection of migration and invasion ability of A549 and SPC-A-1. Scale bar = 100 µm. **C** Plate colony assay of the transfected A549 and SPC-A-1 cells incubated for 3 weeks. **D** The expressions of EMT marker proteins N-cadherin, E-cadherin, Snail and Twist in A549 and SPC-A-1 cells with different IL-17A expression levels were detected by WB. **E** Immunofluorescence analysis of the expression of E-cadherin in A549 andSPC-A-1 with different IL-17A expression levels. Scale bar = 20 µm

IL-17A was highly expressed in NSCLC cells, and to further understand its effect on tumor cells, we chose lung adenocarcinoma cell lines A549 and SPC-A-1 to construct IL-17A knockdown, over-expressed and normal control cells with lentivirus. We found that the wound healing capacities of A549 and SPC-A-1 cells overexpressing IL-17A were found to be significantly increased in the wound healing assay, and the wound healing ability was significantly diminished when IL-17A was knocked down (Fig. 2A). Transwell assays further showed that the number of migrating cells in the IL-17A over-expressed group increased significantly compared to the control group, and the migrating ability decreased after knockdown. Moreover, cells in the over-expressed group were able to degrade the extracellular matrix, and the number of cells crossing to the lower chamber of the transwell was significantly increased, while cells in the knocked-down group were significantly less able to degrade the extracellular matrix (Fig. 2B). Plate colony formation assay is one of the effective methods to detect the proliferation ability of cultured cells. In this assay, we observed that the colony formation ability of A549 and SPC-A-1 cells with high IL-17A expression was significantly enhanced, suggesting that IL-17A could promote tumor cell proliferation (Fig. 2C). All these results supported that IL-17A promoted the proliferation, migration, and invasive ability of NSCLC cells.

The invasiveness and distant metastatic ability of the cells are closely related to the phenotype of the tumor cells, which are mainly manifested by epithelial mesenchymal transition. Thus, we examined the corresponding changes in the expression of EMT-related markers in cells after the alteration of IL-17A expression. We detected epithelial cell marker E-cadherin, mesenchymal cell marker N-cadherin and EMT transcription factor Snail, Twist by western blotting. We observed that the expression of N-cadherin, Snail and Twist increased significantly after overexpression of IL-17A.When IL-17A was knocked down, there was significant increase in expression level of E-cadherin protein(Fig. 2D). We also detected the expression of E-cadherin by immunofluorescence and reconfirmed the negative correlation between IL-17A and E-cadherin expression (Fig. 2E). In summary, IL-17A promotes the proliferation, migration, and invasion of NSCLC cells and the occurrence of EMT, all of which are biological functions that may accelerate tumor progression.

### Blocked of autophagy by IL-17A increased the PD-L1 protein level in NSCLC cells

**Fig. 3:**
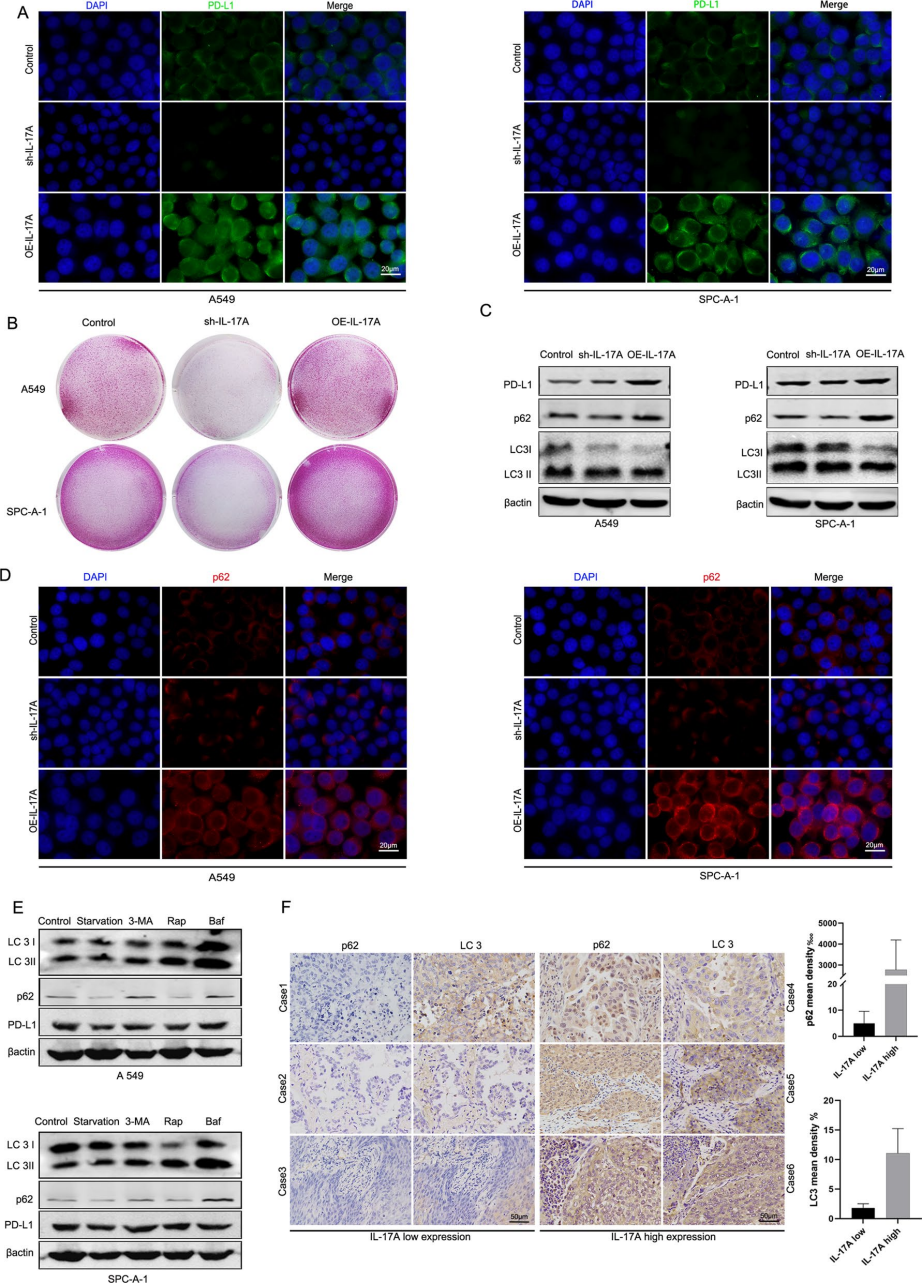
IL-17A blocked autophagy and increased the PD-L1 protein level in NSCLC cells. **A** PD-L1 expression in A549 and SPC-A-1 cells with different IL-17A expression levels were detected by immunofluorescence. Scale bar = 20 µm. **B** A549 and SPC-A-1 cells with different IL-17A expression levels were separately co-cultured with activated Jurkat T cells for 48 h. **C** Western blot analysis of protein expression of PD-L1 and autophagy-related proteins LC3 and p62. **D** Immunofluorescence staining of p62. scale bar = 20 µm. **E** Levels of LC3B I/II, p62 and PD-L1 protein were determined by Western blots in transfected A549 and SPC-A-1 treated with or without starvation, 10 mM of 3-methyladenine (3-MA), 1 nM of bafilomycin A1(Baf) or 20 nM of rapamycin (Rap) for 24 h. **F** Different expression of p62 and LC3 in IL-17A low and high expression groups. Bar = 50 μm

Our clinical samples suggested a positive correlation between IL-17A and PD-L1 expression in tumor cells. To verify this phenomenon in vitro, we separately detected the PD-L1 expression of A549 and SPC-A-1 cells after IL-17A over-expression and knocked-down. Significantly increased PD-L1 in cells over-expressing IL-17A and, conversely, a lower expression of PD-L1 were observed by immunofluorescence (Fig. 3A). To further investigate whether the increased PD-L1 expression has the biological activity to bind PD-1, we co-cultured the transfected cells with CD3/CD28-activated Jurkat T cells. The results demonstrated that the survival rate of tumor cells was markedly improved with high IL-17A expression. This result indicated that the accompanying increased PD-L1 has the effect of reducing the killing ability of Jurkat T cells (Fig. 3B).

Previous studies suggested that the degradation of PD-L1 may be closely related to autophagy, which also plays a dual role in tumorigenesis, progression and drug resistance. To investigate the relationship between IL-17A and autophagy, we examined the transfected cells for autophagy-related proteins. In cells with high expression of IL-17A, both LC3B-II and p62 could be observed to be elevated, suggesting that autophagic flux was blocked, in parallel with elevated expression of PD-L1. In A549 and SPC-A-1 cells with knockdown of IL-17A, LC3B-II and p62 were reduced, suggesting activation of autophagic flux and decreased PD-L1 expression (Fig. 3C). When LC3B-II and p62 were elevated simultaneously, we considered that the cells stayed in autophagosome phase, also called incomplete autophagic process. The observation by immunofluorescence suggested that with the elevation of IL-17A, the intracellular p62 level was significantly elevated (Fig. 3D).

To confirm whether the elevation of PD-L1 was due to blockage of autophagy, we used 3-MA and bafilomycin A1 (Baf) to block autophagy in A549 and SPC-A-1 cells, starvation and rapamycin (Rap) were used to activate autophagy. Results demonstrated that when autophagy was blocked, PD-L1 expression was significantly increased. PD-L1 expression was significantly decreased in A549 and SPC-A-1 cells treated with starvation and Rap (Fig. 3E).

To find out whether similar autophagy exists in clinical NSCLC, we examined the expression of LC3B and p62 in clinicopathological tissues. The difference of LC3 expression in the two groups was not significant, but the positive rate of p62 in the high IL-17A expression group was significantly higher than the low IL-17A expression group (Fig. 3F), indicating the results of in vivo and in vitro experiments were consistent.

### IL-17A blocked autophagy in NSCLC cells via ROS/Nrf2/p62 pathway

**Fig. 4:**
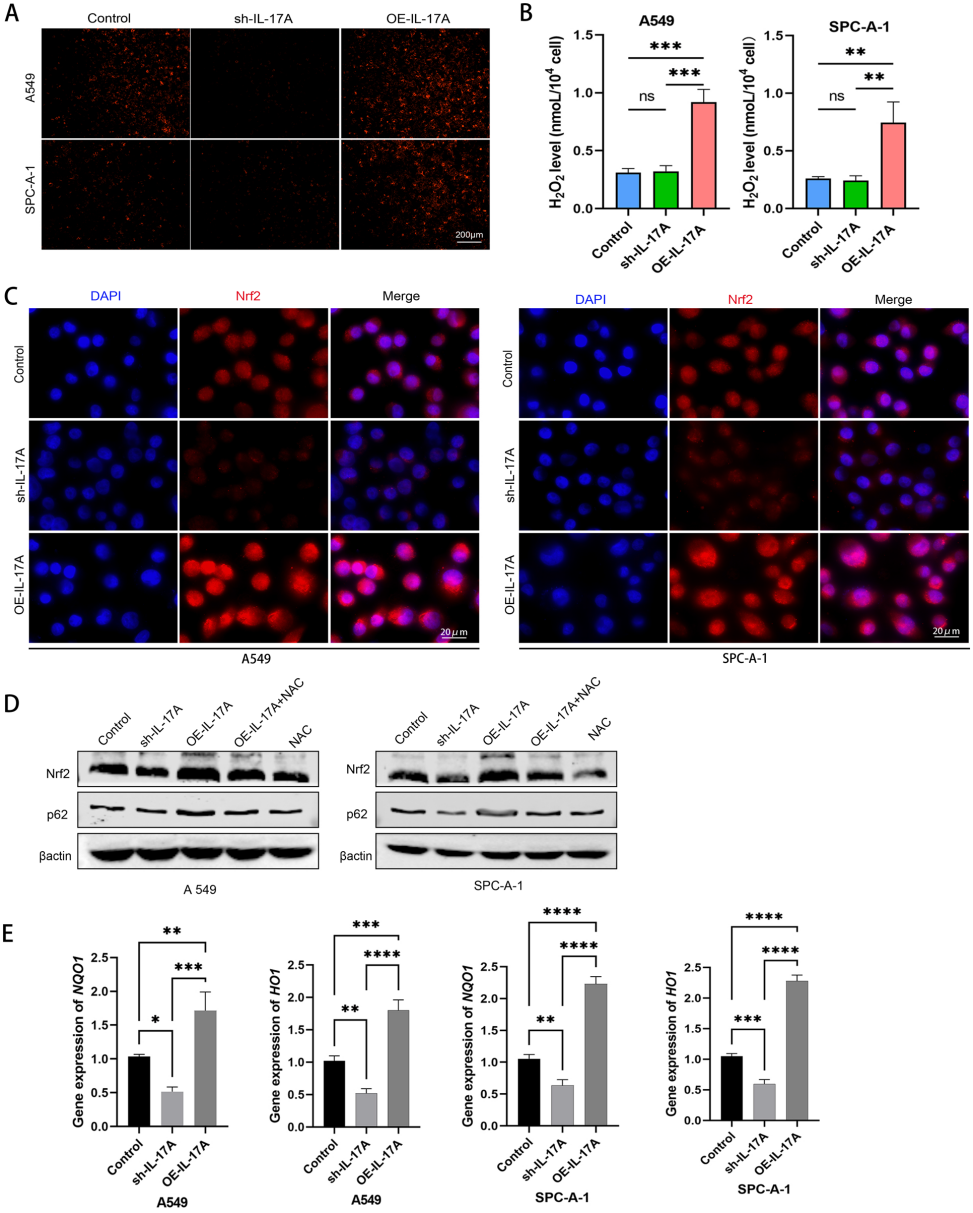
IL-17A blocked autophagy in NSCLC cells via ROS/Nrf2/p62 pathway. **A** The intracellular superoxide anion level was detected by DHE. **B** The intracellular H_2_O_2_ level was measured by microplate method. **C** Immunofluorescence was used to detect the expression and localization of Nrf2 in cells. **D** Protein expression of Nrf2 and p62 at the total protein leve, cells were treated with or without 5 mM of *N*-acetylcysteine (NAC). **E** qPCR detection of *HO1, NQO1* expression.*p < 0.05, **p < 0.01, ***p < 0.001, indicating statistically significant difference

**Fig. 5:**
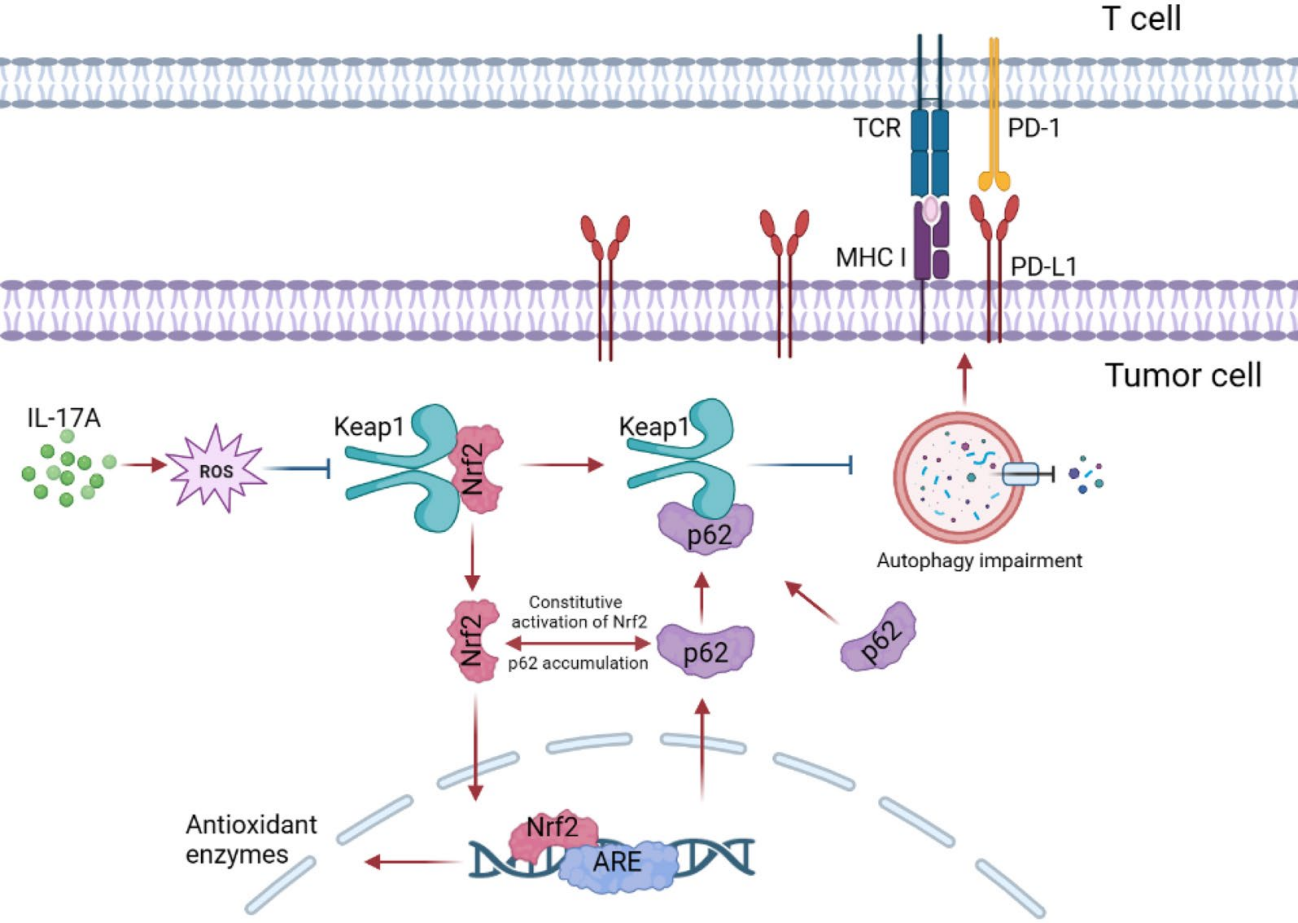
IL-17A induced cellular oxidative stress and activated the Keap1/Nrf2/p62 pathway to block autophagy in NSCLC cells. IL-17A induced intracellular production of low levels of ROS. ROS caused a change in the link site between Keap1 and Nrf2, leading to the dissociation of Keap1 from Nrf2. The dissociated Nrf2 is transferred to the nucleus and binds with ARE to initiate antioxidant reactions. p62 competitively binds Keap1 to Nrf2. When Nrf2 was dissociated from Keap1, the combination of p62 and Keap1 increased, which could chelate Keap1 to autophagosomes. However, excessive accumulation of p62 can cause autophagy dysfunction, resulting in reduced degradation of substrates such as PD-L1 that are degraded through the autophagy pathway

Previous studies suggested that IL-17A can cause oxidative stress, which in turn has a two-sided role in tumor development. Among the currently known reactive oxygen species, superoxide anion and H_2_O_2_ are the main substances. We detected superoxide anion in NSCLC cells with dihydroethidium (DHE) and found that DHE fluorescence intensity was significantly increased in A549 and SPC-A-1 cells over-expressed IL-17A (Fig. 4A). When IL-17A was knocked down, DHE intensity was significantly reduced. The intracellular H_2_O_2_ level also increased significantly with the increase of IL-17A expression which suggested that the elevation of IL-17A expression could increase the intracellular ROS level (Fig. 4B). The increase of intracellular ROS can trigger the antioxidant response, so we examined the antioxidant enzyme nuclear factor erythroid-derived 2-like 2 (Nrf2). We found that the expression of Nrf2 was significantly increased in IL-17A over-expressed cells. Immunofluorescence suggested a tendency for Nrf2 fluorescence to be transferred to the nucleus (Fig. 4C). Moreover, NSCLC cells with elevated Nrf2 expression were accompanied by elevated p62 expression. When we intervened the cells with antioxidant NAC, we found that antioxidation led to a decrease in Nrf2 and p62 levels, which could antagonise the effects of IL-17A overexpression (Fig. 4D). Nrf2 is activated and translocated into the nucleus, which increase the expression of antioxidant gene HO1 and NQO1 (Fig. 4E). We hypothesize that this phenomenon is caused by the release of Nrf2 from Keap1 when NSCLC cells are exposed to reactive oxygen species, and then the dissociated Nrf2 is not degraded by the proteasome but transferred to the nucleus. p62 binds Keap1 competitively with Nrf2. Once Keap1 dissociates from Nrf2, p62 binding to Keap1 could be increased, leading to an intracellular accumulation of p62, which would cause incomplete autophagy. Then the degradation process of PD-L1 by autophagy pathway was inhibited. As a result, the expression level of PD-L1 would be increased (Fig. 5).

### Blocking IL-17A suppressed tumor growth in vitro but reduced the efficacy of anti-PD-1 therapy in vivo by decreasing PD-L1 protein expression

**Fig. 6:**
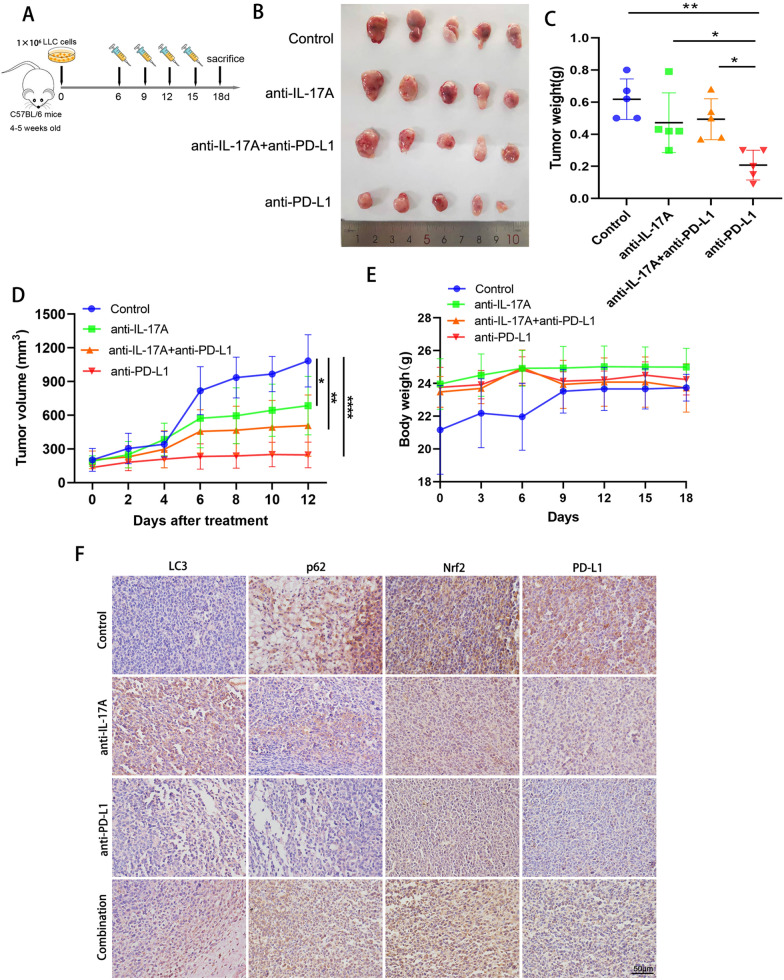
Blocking IL-17A suppressed tumor growth in vitro but reduced the efficacy of anti-PD-1 therapy in vivo by decreasing PD-L1 protein expression. **A** Schematic diagram of the establishment of animal subcutaneous tumor model. **B–D** Tumor volume and weight of mice in control group, anti-IL-17 monoclonal antibody treatment group, anti-PD-L1 monoclonal antibody treatment group, and anti-IL-17A combined with anti-PD-L1 treatment group. **E** Changes in body weight of mice in 4 groups after drug intervention. **F** Immunohistochemistry was used to detect the expression levels of LC3, p62, Nrf2 and PD-L1 in tumor tissues. *p < 0.05, **p < 0.01, ***p < 0.001, indicating statistically significant difference

To explore the therapeutic effects of blocking IL-17A in vivo and understand its relationship with immunotherapy, we established a subcutaneous tumor model in mice. LLC was inoculated into the right flank of C57BL/6 mice and respectively treated with PBS, anti-IL-17 mAb, anti-PD-L1 mAb and anti-IL-17A combined with anti-PD-L1 therapy on days 6, 9, 12, and 15. Mice were sacrificed on day 18 and tumor tissue was collected (Fig. 6A). Compared with the control group, the tumor size and weight of anti-IL-17A and anti-PD-L1 treatment groups were reduced to some extent, the growth rate was significantly slowed down, but the efficacy of the combined treatment was inferior to that of anti-PD-L1 monotherapy (Fig. 6B–D). Meanwhile, we monitored the body weight of mice during the experiment and found that the change in body weight was not significant among all four groups, suggesting that the drug was tolerable and safe for mice (Fig. 6E). To confirm the consistency of the effects of IL-17A in vitro and in vivo, we examined the expression of p62 and Nrf2 in tumor tissues, which were decreased. And the expression of PD-L1 was significantly reduced in the tumor tissues of the anti-IL-17A mAb and combination treatment groups (Fig. 6F).

## Discussion

IL-17A is considered to be a prevalent cytokine in the tumor microenvironment and plays a dual role in tumor growth and elimination. IL-17 regulates immune responses to microorganisms and autoimmune responses, balancing cytotoxicity and tolerogenic immunity, while also contributing to chronic inflammation. IL-17 causes immunosuppression associated with chronic inflammatory responses that may be exploited by cancer and undermine the effectiveness of immune checkpoint inhibitors [24]. Consistent with previous findings, we observed that IL-17A was generally elevated in NSCLC tumor tissues and positively correlated with PD-L1 expression. In vitro, IL-17A promoted the proliferation, invasion, migration and EMT of NSCLC A549 and SPC-A-1 cells. Meanwhile, tumor cells with high IL-17A expression can reduce cellular PD-L1 degradation by blocking autophagy, contributing to sustained high PD-L1 expression in tumor cells. We further found that this effect of IL-17 was mediated by promoting ROS production, Nrf2 activation and p62 accumulation. In vivo experiments, blocking IL-17A can slow down tumor growth. However, when both IL-17A and PD-L1 were simultaneously inhibited, tumor cells expressed less PD-L1 and anti-PD-L1 losed its target, leading to a decrease therapeutic effect. Based on our experimental results, we hypothesized that IL-17A promoted NSCLC development by prompting the conversion of tumor cells to a pro-tumor growth phenotype and increasing PD-L1 expression. IL-17A is a potent pro-inflammatory cytokine. Chronic IL-17A activity-mediated production of inflammatory mediators mobilizes immunosuppression and recruits myeloid cells, creating an immune microenvironment suitable for tumor growth [25]. We found that IL-17A was elevated in squamous carcinoma and adenocarcinoma. IL-17A was positively correlated with the expression of PD-L1, which is in line with previous findings that IL-17A was found to induce cellular expression of PD-L1 in hepatocellular carcinoma stem cells, colorectal cancer, estrogen receptor-negative breast cancer, and cholangitis. Activation of IL-17A signaling promotes self-renewal and immune escape of tumor stem cells [23, 26–28]. Lung cancer is closely associated with chronic inflammation, and previous studies have provided evidence that local lung flora can promote inflammation and tumor cell proliferation by activating lung-resident γδT cells and releasing specific immune mediators such as IL-17A [29]. Airborne particulate matter such as PM2.5 also regulates tumor cell proliferation, metastasis and EMT by promoting the expression of IL-17A in lung cancer tissues [30]. The IL-17A that causes these inflammation-associated malignancies can be secreted by Th17 lymphocytes or by the tumor cells themselves [31, 32]. To understand the role of intracellular IL-17A in tumor cells. We constructed IL-17A knockdown and over-expressed isotypes in A549 and SPC-A-1 cell lines by lentivirus to better cover its intracellular and paracrine roles. The experimental results suggested that endogenous IL-17A could stably promote lung cancer cell migration, invasion and EMT, while knockdown of IL-17A significantly decreased these abilities of tumor cells.

Autophagy is one of the most important survival mechanisms of tumor cells, which helps tumor cells adjust and adapt to an unfavorable environment and thus escape from immune surveillance, eventually promoting tumor growth. A study found that inhibition of autophagy increased PD-L1 expression in gastric cancer cells. Adjusting autophagy can influence the therapeutic effect of PD-L1 blockade [19]. We demonstrated that overexpressed IL-17A blocked the pathway of autophagy and the selective autophagy protein p62 was accompanied by an increase in PD-L1 and significantly reduced the killing capacity of Jurket T cells. In transfected A549 and SPC-A-1 cells, intervening in different stages of autophagy can affect the expression of cellular PD-L1, suggesting that the regulation of autophagy can affect PD-L1 expression. Monoclonal antibodies against IL-17A, such as Ixekizumab, Secukinumab, and Bimekizumab have been widely used clinically in immune-mediated inflammatory diseases such as psoriasis, psoriatic arthritis, and axial spondyloarthritis, showing high selectivity and safety [33–35]. Precise targeting IL-17A may efficiently modulate PD-L1 expression in lung cancer cells. Previous studies have shown that modulation of PD-L1 can control cancer immune surveillance, and a general and stable PD-L1 regulatory mechanism should be found to improve the response rate to immune checkpoint inhibitors [36].

We found that IL-17A stably caused elevated levels of p62 protein in A549 and SPC-A-1 cells, the latter being defined as a selective autophagy adaptor protein, which can act as a major regulator of Nrf2, mTORC1, and NF-κB signaling pathway activation, linking p62 to oxidative defense system, nutrient sensing, and inflammation, respectively [37]. Sustained expression of IL-17A in chronically inflamed tissues induces elevated expression of superoxide and hydrogen peroxide in arterial smooth muscle cells [38]. Stimulation of gastric cancer cells with recombinant human IL-17A protein promotes cell growth, ROS generation and increased stemness of tumor stem cells [39]. Therefore, we hypothesized that there is an IL-17A/ROS/p62 axis regulatory mechanism in NSCLC. The assay revealed that IL-17A induced a mild increase in H_2_O_2_ and superoxide anion production by tumor cells. It is known that ROS can activate Nrf2 through the classical pathway, and Keap1 dissociates from Nrf2, binds to p62 and is transferred to the autolysosome [40]. A study on anti-vascular drugs found that Apatinib induced autophagy and apoptotic cell death in NSCLC cells by promoting ROS production and inhibiting the expression of Nrf2 and p62 [41]. Combined with our experimental results, it suggested that IL-17A blocking autophagy may be regulated by both classical and non-classical pathways of Nrf2, which promotes Keap1 and p62 combination, thus affecting the p62-mediated autophagy.

The role of IL-17A in NSCLC patients treated with immune checkpoint inhibitors remains unclear. In a multicenter retrospective analysis, when 56 NSCLC patients with an AID received a PD-L1 inhibitor, 23% of them had an exacerbation of AID, and 38% of patients developed irAE [42]. Moreover, there was a case report that patients with metastatic colon cancer who experienced severe psoriasis exacerbation (previously mild) after three cycles of pembrolizumab (Anti-PD-1). The cancer progressed after administration of secukinumab (a human circulating interleukin-17A monoclonal antibody) for psoriasis while continuing to receive pembrolizumab for another two cycles [43]. IL-17A promotes tumorigenesis by upregulating PD-L1 expression in hepatocellular stem cells, helping cancer cells to self-renew and escape immune attack [27]. PD-L1 functions differently in tumors with different immunogenicity. In tumors with high immunogenicity, it is not only a marker of suppressive immune microenvironment, but also a key mechanism that attenuates the cytotoxicity of CD8^+^ T cells [44]. In our in vivo experiments, it can be found that blocking IL-17A alone can inhibit tumor growth to some extent, but when combined with anti-PD-L1 drugs, cancer tissue expression of PD-L1 is reduced, which makes anti-PD-L1 drugs lose their targets and the anti-tumor effect is diminished. Therefore, we hypothesize that the efficacy of immune checkpoint inhibitors may be compromised in lung cancer patients with AID following the use of IL-17A monoclonal antibody.

## Conclusion

In conclusion, our results show that IL-17A promotes NSCLC cell proliferation and increases PD-L1 expression in tumor cells by reducing PD-L1 degradation through inhibition of autophagy, creating a suppressive immune microenvironment. Antagonizing IL-17A alone may inhibit tumor progression, but in combination with immunotherapy may alter the tumor microenvironment to affect efficacy.

## Data Availability

The datasets supporting the conclusions of this article are included within the article.
